# Introduction to sonosurgery

**DOI:** 10.1186/1878-5085-5-S1-A66

**Published:** 2014-02-11

**Authors:** Zbigniew Pilecki, Grzegorz Pilecki, Rostyslav Bubnov

**Affiliations:** 1 Sports Medicine Center in Zabrze, Poland; 2 Clinical Hospital “Pheophania” of State Affairs Department, Kyiv, Ukraine

## Introduction

The dynamic development of minimally invasive techniques in surgery was based on access to imaging equipment, endoscopic sets and surgeons’ huge passion. It is a great imagination, responsibility and courage of surgeons was the driving force increasing use of minimally invasive techniques. Surgery as discipline is developing to the direction for minimizing damage during the intervention. With the appearance of opportunities imaging in real time conditions were created for the performance of invasive procedures in ultrasound imaging [1,2].

## Sonosurgery

Sonosurgery is a collection of minimally invasive surgical techniques performed with continuous ultrasound imaging and the use of endoscopic tools. It’s a surgical discipline and requires compliance of aseptic and medical art conditions and should be performed in the operating unit by experienced personnel. By medical art we understand mastery in surgical techniques and ability to perform ultrasound examination by a physician. However, the simplicity and minimal tissue trauma during sonosurgical procedures will lead to execute them in operating room similar to procedures in interventional ultrasonography [3]. Sonosurgical techniques are techniques performed in the conventional surgery and orthopedics, but the use of ultrasound equipment can reduce operating approach and reduce invasive procedure to the affected tissue.

## Stages in sonosurgical procedures

We perform sonosurgery successfully for three years systematically increasing range of operations. All procedures in sonosurgery have their steps, which we will present: I. Performing of a sonotopogram; II. Preparations techniques: 1) Using liquid (needle technique); 2) Using tools; 3) Using electrotools; 4) Balloon techniques; 5) Mixed techniques; III. Endoscopic tools; IV. Sewing. Operations are performed in one-day procedure. The cost of ultrasound imaging amortization is low. Using ultrasound imaging, we can often control the course of healing of the operated structures. This is not a threat to the patient and the costs are also low.

## Conclusions

Use of ultrasound imaging allows a very precise, atraumatically and aseptically performing of surgery. Sonosurgical technique has all the characteristics of minimal invasive treatment: good cosmetic result, minimal tissue trauma, low pain, the possibility of taking early rehabilitation and a very good final result. The total cost of sonosurgical treatment, compared to procedures performed in classical techniques is low, because of minimal amount of materials used for the treatment, the number of staff and the way of anesthesia.

## Outlook and expert recommendations

It is recommended to promote programs to implement sonosurgical techniques, building safe and modern hospital [4]. For support appropriate level of hardware and experienced surgical team for high level of treatment outcome and patient safety is recommended:

• to start programs to make the ultrasound devices to be available in every operating room;

• to organize interdisciplinary educational programs for surgeons, radiologists, and nurse staff;

• to initiate comparative studies to establish science based treatment algorithms.
